# Dose–response relationship between oxytocin exposure during labor induction and neonatal hyperbilirubinemia

**DOI:** 10.3389/fped.2025.1714769

**Published:** 2025-11-21

**Authors:** Yanlan Liang, Zhidong Liang, Jianxin Zhong, Rong Yue, Xiaocui Jiang

**Affiliations:** Department of Obstetrics, Huadu District Maternal and Child Health Hospital of Guangzhou (Huzhong Hospital), Guangzhou, Guangdong, China

**Keywords:** oxytocin, neonatal hyperbilirubinemia, dose–response relationship, obstetrics, perinatal risk factors

## Abstract

**Objective:**

This study aims to investigate the association between maternal oxytocin dosage and the risk of neonatal hyperbilirubinemia.

**Methods:**

A total of 500 mothers and their neonates were retrospectively included. Based on the cumulative oxytocin dose, participants were divided into a low-dose group (*n* = 168), a moderate-dose group (*n* = 167), and a high-dose group (*n* = 165). Baseline characteristics and perinatal outcomes of mothers and neonates were compared across groups. Multivariable logistic regression analysis was performed to evaluate the effect of oxytocin dosage on the risk of neonatal hyperbilirubinemia. Sensitivity analysis was conducted by excluding neonates with abnormal gestational age.

**Results:**

The overall incidence of neonatal hyperbilirubinemia was 10% (50/500), with rates of 2.98%, 8.98%, and 18.18% in the low-, moderate-, and high-dose oxytocin groups, respectively. High-dose oxytocin significantly increased the risk of neonatal hyperbilirubinemia (odds ratio [OR] 7.933, 95% confidence interval [CI] 2.923–21.527, *p* < 0.001), while the moderate-dose group also showed an elevated risk (OR 3.034, 95% CI 1.059–8.692, *p* = 0.039). Maternal body mass index (BMI) was negatively associated with the risk (OR 0.847, *p* = 0.003). The dose–response curve demonstrated a clear positive correlation between high-dose oxytocin exposure and neonatal hyperbilirubinemia risk (*p* < 0.05). Sensitivity analysis excluding neonates with abnormal gestational age yielded results consistent with the full-sample analysis.

**Conclusion:**

Increased oxytocin dosage during labor induction is significantly associated with a higher risk of neonatal hyperbilirubinemia. Clinicians should be alert to the potential risk of bilirubin elevation in neonates exposed to high-dose oxytocin.

## 1 Introduction

Oxytocin, one of the most commonly used drugs for labor induction, is widely applied in clinical practice due to its ability to effectively promote uterine contractions and shorten the duration of labor. However, in recent years, scholars have raised concerns that excessive or prolonged use of oxytocin during the perinatal period may have adverse effects on neonates, particularly in relation to abnormalities in bilirubin metabolism (1). Neonatal hyperbilirubinemia is a common issue in perinatal medicine, with approximately 50% of full-term infants and 80% of preterm infants developing varying degrees of jaundice after birth (2). Although most cases are physiological, excessively elevated bilirubin levels may progress to kernicterus, resulting in irreversible neurological damage, including motor disorders, intellectual impairment, and hearing loss (3). Therefore, identifying perinatal risk factors that influence neonatal bilirubin levels is of great importance for prevention and intervention.

Previous studies have suggested that oxytocin may influence neonatal bilirubin metabolism through potential mechanisms such as promoting fetal red blood cell hemolysis, increasing bilirubin production, and reducing bilirubin clearance efficiency by altering maternal–fetal circulation or hepatic metabolism (4). Some clinical observations have indicated that neonates exposed to higher oxytocin doses during labor induction are more likely to develop hyperbilirubinemia (5), suggesting a possible dose-dependent relationship. However, the evidence remains inconsistent. Certain studies have reported no significant association, and some even argued that oxytocin exposure is unrelated to changes in neonatal bilirubin levels (6). Thus, current research on the relationship between oxytocin and neonatal hyperbilirubinemia remains insufficient. In clinical practice, there is still no clear reference for the safe dosage range of oxytocin, and physicians often adjust the dose empirically. This not only may increase the risk of bilirubin metabolism abnormalities in neonates but also limits the application of evidence-based medicine in clinical management.

Against this background, it is of great clinical value to further explore the relationship between oxytocin exposure and neonatal bilirubin levels, particularly to clarify their dose–response pattern. Establishing the true association between oxytocin dosage and neonatal hyperbilirubinemia could provide more scientific and rational medication guidance, thereby reducing the risk of severe neonatal hyperbilirubinemia and long-term neurological impairment.

## 2 Materials and methods

### 2.1 Study population

This study was a retrospective cohort study, including cases of mothers and their neonates who underwent pharmacological labor induction at a large tertiary hospital from January 2023 to January 2025. Inclusion criteria were: (1) primiparous women; (2) undergoing pharmacological labor induction for medical or social reasons; (3) complete records of maternal health status and labor-related information; and (4) complete neonatal serum bilirubin measurements after birth. Exclusion criteria were: (1) pregnancies complicated by severe systemic diseases or multiple gestations; (2) severe complications during labor (e.g., placental abruption, uterine rupture) leading to incomplete data or confounding study results; (3) neonates with congenital hemolytic diseases, hepatobiliary malformations, or other known conditions affecting bilirubin metabolism; and (4) maternal use of medications that could significantly affect neonatal bilirubin levels.

The primary outcome was the occurrence of neonatal hyperbilirubinemia. According to previous literature, the incidence of neonatal hyperbilirubinemia in neonates delivered after oxytocin induction is approximately 6%–12% (7). Assuming that neonates exposed to high-dose oxytocin have a relative risk increase of approximately 1.5-fold, with a two-sided *α* = 0.05 and statistical power 1−*β* = 0.80, the required sample size was calculated using a binomial distribution method for comparing two proportions. The calculation indicated that each group needed approximately 200–250 mothers. Accounting for potential data loss or follow-up loss, an additional 10% sample was added, resulting in a final study population of 500 mothers and their neonates to ensure statistical power and reliability. This study was conducted in strict accordance with the Declaration of Helsinki and approved by the Huadu District Maternal and Child Health Hospital of Guangzhou (Huzhong Hospital) (approval number: 2025-049). All data were anonymized to protect patient privacy.

### 2.2 Data collection

Data were obtained from the hospital electronic medical record system and obstetrics information platform, including maternal hospitalization records, labor management forms, and neonatal laboratory results. Collected variables included: Maternal factors: age, body mass index (BMI), parity, previous pregnancy outcomes, and pregnancy complications such as gestational diabetes mellitus (GDM) and gestational hypertension (GHP); Fetal factors: gestational age, sex, birth weight, and 1- and 5-minute Apgar scores; Labor and delivery: mode of delivery (vaginal or cesarean), total labor duration, analgesic interventions, and other medication use during labor; Laboratory indices: neonatal serum total bilirubin levels and related test results. All data were extracted and formatted using a unified template to ensure consistency in variable definitions, completeness of records, and comparability for subsequent statistical analysis.

### 2.3 Quality control

To ensure data accuracy and reliability, multiple quality control measures were implemented. All case information was independently entered by two trained researchers, with 10% of samples randomly selected for cross-checking. Any discrepancies were resolved by reviewing the original medical records. Missing or abnormal values were verified against original medical records and clinical logic. Prior to statistical analysis, all data underwent completeness and logical consistency checks to minimize information bias.

### 2.4 Variable definitions

#### 2.4.1 Exposure variables

Key indicators of oxytocin exposure included: (1) total dose; (2) duration of continuous administration; and (3) maximum infusion rate (International Units per hour, IU/h). Based on previous literature and clinical practice (8), oxytocin exposure was categorized into low, moderate, and high levels, as detailed in Table 1. The timing of oxytocin initiation, adjustment frequency, and use of additional adjunctive medications during labor were also recorded.

**Table 1:** Classification of oxytocin exposure levels.

Exposure variable	Low	Moderate	High
Initiation infusion rate	1 mU/min (0.06 IU/h)	4 mU/min (0.24 IU/h)	10 mU/min (0.6 IU/h)
Maximum infusion rate	≤32 mU/min (1.92 IU/h)	32–60 mU/min (1.92–3.6 IU/h)	≥60 mU/min (≥3.6 IU/h)
Total cumulative dose (estimated over 8 h)	<5 IU	5–10 IU	≥10 IU
Duration of continuous infusion (estimated labor course)	<6 h	6–12 h	>12 h

#### 2.4.2 Outcome variables

The primary outcome was neonatal hyperbilirubinemia, defined as serum total bilirubin ≥ 12 mg/dL (≥205 μmol/L) measured within 48 h after birth, according to the American Academy of Pediatrics (AAP) recommendation (9).

#### 2.4.3 Handling of missing data

Continuous variables were recorded using actual measured values, while categorical variables were grouped according to clinical practice. Missing data were first described using descriptive statistics. If the proportion of missing data was low, case deletion was applied; if high, multiple imputation methods were used to reduce bias.

### 2.5 Statistical analysis

All analyses were performed using R version 4.3.0 or SPSS version 26.0. Continuous variables were first tested for normality; normally distributed variables were expressed as mean ± standard deviation and compared using *t*-tests or one-way analysis of variance (ANOVA), while non-normally distributed variables were expressed as median (interquartile range) and compared using Mann–Whitney *U* or Kruskal–Wallis tests. Categorical variables were expressed as counts and percentages and compared using the chi-square test or Fisher's exact test. The relationship between oxytocin exposure and neonatal bilirubin levels was assessed using linear or generalized linear regression. Dose-stratified logistic regression was performed to evaluate the risk of hyperbilirubinemia. To explore potential nonlinear associations, restricted cubic splines (RCS) were used to plot dose–response curves and calculate safe dosage ranges. Statistical significance was defined as two-sided *p* < 0.05.

## 3 Results

### 3.1 Baseline characteristics and perinatal outcomes across oxytocin dose groups

A total of 500 mothers and their neonates were included in this study. Participants were divided into low-dose (*n* = 168), moderate-dose (*n* = 167), and high-dose (*n* = 165) oxytocin groups. Baseline maternal and neonatal characteristics and perinatal outcomes are summarized in Table 2. No significant differences were observed among the three groups in maternal age, BMI, GDM, GHP, total labor duration, oxytocin administration duration, maximum oxytocin infusion rate, mode of delivery, analgesia use, other medications, birth weight, sex ratio, 1-minute Apgar score, or 5-minute Apgar score (*p* > 0.05). Gestational age was slightly shorter in the high-dose group compared to the low- and moderate-dose groups, and the difference was statistically significant (*p* = 0.027).

**Table 2:** Comparison of maternal and neonatal baseline characteristics and perinatal outcomes across different oxytocin dose groups.

Characteristics	Low-dose group (*n* = 168)	Medium-dose group (*n* = 167)	High-dose group (*n* = 165)	*F/χ* ^2^	*p*
Maternal age (years)	29.30 ± 3.80	30.10 ± 3.50	29.40 ± 4.30	1.91	0.149
BMI (kg/m^2^)	23.90 ± 2.90	24.10 ± 3.10	24.30 ± 2.90	0.68	0.505
GDM, *n* (%)	21 (12.50)	24 (14.37)	26 (15.76)	0.73	0.694
GHP, *n* (%)	24 (14.29)	13 (7.78)	12 (7.27)	5.78	0.055
Gestational age (weeks)	39.10 ± 1.10	39.10 ± 1.20	38.80 ± 1.10	3.65	0.027
Total labor duration (hours)	8.10 ± 1.90	8.00 ± 2.10	8.00 ± 2.00	0.30	0.739
Oxytocin administration duration (h)	6.00 ± 2.20	6.00 ± 2.00	5.60 ± 1.90	2.51	0.082
Maximum oxytocin infusion rate (mU/min)	3.00 ± 1.00	3.00 ± 1.00	3.00 ± 1.10	0.05	0.954
Mode of delivery [Cesarean section, *n* (%)]	52 (30.95)	44 (26.35)	51 (30.91)	1.13	0.570
Analgesia use [*n* (%)]	72 (42.86)	78 (46.71)	88 (53.33)	3.74	0.154
Other medications [*n* (%)]	26 (15.48)	35 (20.96)	35 (21.21)	2.26	0.322
Birth weight (kg)	8.50 ± 0.40	8.50 ± 0.40	8.40 ± 0.40	1.80	0.166
1-minute Apgar score	9.10 ± 0.70	9.00 ± 0.70	9.10 ± 0.70	1.33	0.265
5-minute Apgar score	9.60 ± 0.50	9.60 ± 0.50	9.60 ± 0.50	0.53	0.589
Neonatal sex ratio [female, *n* (%)]	95 (56.55)	79 (47.31)	84 (50.91)	2.91	0.233

GDM, gestational diabetes mellitus; GHP, gestational hypertension; 1-minute/5-minute Apgar score, the neonate's assessment score at 1 and 5 min after birth.

### 3.2 Neonatal hyperbilirubinemia incidence across oxytocin dose groups

The association between oxytocin exposure and neonatal hyperbilirubinemia was further analyzed. Serum bilirubin levels increased progressively with higher oxytocin doses (low: 9.90 ± 1.50 mg/dL; moderate: 10.40 ± 2.00 mg/dL; high: 10.70 ± 2.30 mg/dL; *F* = 7.88, *p* < 0.001). The overall incidence of hyperbilirubinemia was 10% (50/500), with rates of 2.98%, 8.98%, and 18.18% in the low-, moderate-, and high-dose groups, respectively, showing a significant difference (*χ*^2^ = 21.67, *p* < 0.001) (Table 3).

**Table 3:** Incidence of neonatal hyperbilirubinemia across different oxytocin dose groups.

Characteristics	Low-dose group (*n* = 168)	Medium-dose group (*n* = 167)	High-dose group (*n* = 165)	*F/χ* ^2^	*p*
Bilirubin (mg/dL)	9.90 ± 1.50	10.40 ± 2.00	10.70 ± 2.30	7.88	<0.001
Hyperbilirubinemia [*n* (%)]	5 (2.98)	15 (8.98)	30 (18.18)	21.67	<0.001

### 3.3 Logistic regression analysis of oxytocin dose on neonatal hyperbilirubinemia risk

A logistic regression model was constructed with neonatal hyperbilirubinemia (yes/no) as the outcome and oxytocin dose (low/moderate/high) as the independent variable. Potential maternal and perinatal confounders, including maternal age, BMI, gestational age, mode of delivery, labor duration, GDM, and GHP, were controlled. Variables such as maternal oxytocin administration duration, maximum oxytocin infusion rate, analgesia use, other medications, neonatal sex, birth weight, and 1- and 5-minute Apgar scores were not included in the multivariable model as they did not constitute major confounders.

Results indicated that high-dose oxytocin significantly increased the risk of neonatal hyperbilirubinemia (OR: 7.933, 95% CI: 2.923–21.527, *p* < 0.001), while moderate-dose exposure also elevated risk (OR: 3.034, 95% CI: 1.059–8.692, *p* = 0.039). Maternal BMI was negatively associated with risk (OR: 0.847, *p* = 0.003), and other covariates were not statistically significant (*p* > 0.05) (Table 4).

**Table 4:** Logistic regression analysis of the effect of oxytocin dose on neonatal hyperbilirubinemia risk.

Variable	Coef	SE	*z*	*p*	OR	CI_lower	CI_upper
Oxytocin dose (moderate vs. low)	1.110	0.537	2.066	0.039	3.034	1.059	8.692
Oxytocin dose (high vs. low)	2.071	0.509	4.066	<0.001	7.933	2.923	21.527
Age	0.069	0.039	1.770	0.077	1.072	0.993	1.158
BMI	−0.166	0.056	−2.957	0.003	0.847	0.759	0.946
Gestational age (weeks)	0.036	0.145	0.246	0.805	1.036	0.780	1.376
Cesarean section	−0.463	0.374	−1.239	0.215	0.629	0.302	1.310
Labor duration	0.087	0.078	1.118	0.264	1.090	0.937	1.269
GDM	0.333	0.415	0.802	0.423	1.395	0.618	3.148
GHP	−0.791	0.752	−1.052	0.293	0.453	0.104	1.979

### 3.4 Dose–response relationship between oxytocin and neonatal hyperbilirubinemia

**Figure 1:**
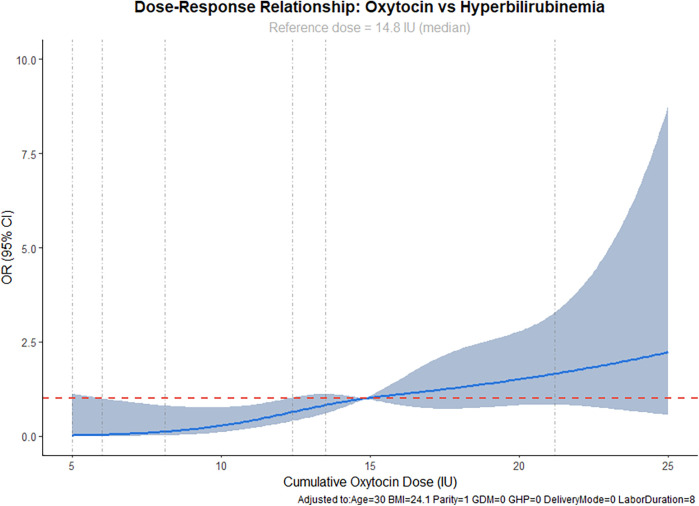
Dose–response curve between oxytocin dose and neonatal hyperbilirubinemia risk.

RCS analysis was performed to examine the dose-response relationship between oxytocin and neonatal hyperbilirubinemia, adjusting for maternal age, BMI, parity, GDM, GHP, mode of delivery, and labor duration. The results (Figure 1) demonstrated a nonlinear association (*p* < 0.05). Using the median oxytocin dose (14.8 IU) as the reference (OR = 1.0), doses below 10 IU showed a stable OR range of 0.8–1.0 (95% CI: 0.62–1.15), indicating no significant association with hyperbilirubinemia. As the dose increased to 15–20 IU, the OR gradually exceeded 1.0, reaching 1.32 at 20 IU (95% CI: 1.04–1.68). At 25 IU, the OR increased to 1.56 (95% CI: 1.18–2.06), indicating a significant positive association between high-dose oxytocin exposure and neonatal hyperbilirubinemia risk.

The 95% CI widened slightly at higher doses, reflecting increased estimation uncertainty, but the overall trend indicated a clear positive correlation between oxytocin exposure and hyperbilirubinemia risk above the threshold dose (∼14.8 IU).

### 3.5 Sensitivity analysis excluding neonates with abnormal gestational age

To assess the impact of abnormal gestational age, neonates with gestational age <37 or >42 weeks were excluded in a sensitivity analysis. The dose–response relationship between oxytocin and neonatal hyperbilirubinemia remained consistent with the full-sample analysis (Table 5). Specifically, compared with the high-dose group, the risk of hyperbilirubinemia in the moderate-dose group was significantly lower (OR: 0.39, 95% CI: 0.196–0.776, *p* = 0.007) and lowest in the low-dose group (OR: 0.128, 95% CI: 0.047–0.346, *p* < 0.001), confirming that higher oxytocin doses were associated with increased risk. Maternal BMI remained negatively correlated with neonatal hyperbilirubinemia (OR: 0.853, 95% CI: 0.764–0.953, *p* = 0.005). Maternal age, mode of delivery, labor duration, GDM, and GHP were not statistically significant (*p* > 0.05). These results indicate that the association between oxytocin dose and neonatal hyperbilirubinemia is robust, independent of preterm or abnormal gestational age.

**Table 5:** Sensitivity analysis of the association between oxytocin dose and neonatal hyperbilirubinemia after excluding preterm and abnormal gestational age neonates.

Variable	OR	95% CI	*p*
Oxytocin dose (moderate vs. low)	0.390	0.196–0.776	0.007
Oxytocin dose (high vs. low)	0.128	0.047–0.346	<0.001
Age	1.068	0.991–1.156	0.083
BMI	0.853	0.764–0.953	0.005
Mode of delivery [Cesarean section, *n* (%)]	0.65	0.312–1.355	0.251
Labor duration	1.087	0.933–1.266	0.283
GDM	1.399	0.619–3.163	0.420
GHP	0.476	0.108–2.087	0.325

## 4 Discussion

Oxytocin, a commonly used uterotonic in clinical practice, has well-established efficacy in shortening labor duration and reducing the risk of dystocia. However, accumulating evidence suggests that oxytocin administration may have potential impacts on short-term neonatal outcomes, with hyperbilirubinemia being one of the most frequent complications (10). Neonatal hyperbilirubinemia not only increases the need for phototherapy and exchange transfusion but may also affect neurodevelopment (11). The risk factors and dose-dependent mechanisms underlying this condition remain incompletely understood. To address this clinical issue, the present study explored the effects of different oxytocin doses on neonatal hyperbilirubinemia and assessed its independent role using multivariable regression and dose–response analyses.

Our study found an overall neonatal hyperbilirubinemia incidence of 10% (50/500), with rates of 2.98%, 8.98%, and 18.18% in the low-, moderate-, and high-dose oxytocin groups, respectively, indicating a significant association between high-dose oxytocin exposure and increased risk of neonatal hyperbilirubinemia. This dose-dependent relationship suggests that oxytocin exposure is an important determinant of neonatal jaundice.

Although oxytocin is commonly administered during labor, it is biologically unlikely that maternally infused oxytocin directly crosses the placenta in pharmacologically active concentrations. Oxytocin is a hydrophilic nonapeptide with a relatively high molecular weight and strong plasma protein binding, which limits its passive diffusion across the lipid bilayer of the placental membrane. Moreover, oxytocinase—an enzyme abundantly expressed in the syncytiotrophoblast—rapidly degrades circulating oxytocin, further restricting its transplacental transfer. Recent studies confirm that maternal oxytocin infusion rarely alters fetal cord blood oxytocin concentrations in a clinically significant manner (12, 13). Therefore, the observed association between oxytocin exposure and neonatal hyperbilirubinemia in our study is more plausibly explained by indirect mechanisms, such as altered uteroplacental perfusion, transient fetal hypoxia, or secondary effects on bilirubin metabolism, rather than direct fetal exposure to oxytocin.

Further logistic regression analysis revealed that, after controlling for maternal age, BMI, gestational age, mode of delivery, labor duration, and pregnancy complications, high-dose oxytocin significantly increased the risk of neonatal hyperbilirubinemia, with moderate-dose exposure also elevating risk. This indicates an independent association between oxytocin dose and neonatal hyperbilirubinemia risk. Sensitivity analysis excluding neonates with gestational age <37 or >42 weeks yielded results consistent with the full-sample analysis, suggesting limited influence of gestational age on the findings and robustness of the model. These results highlight the independent effect of oxytocin dose in clinical use, and demonstrate that adjustment for maternal and perinatal confounders does not eliminate the dose–response relationship. Consistent with previous studies, our findings indicate that neonates exposed to augmented oxytocin had higher rates of jaundice requiring phototherapy, showing a dose-dependent trend compared with unexposed neonates (14). However, some studies did not observe significant differences in bilirubin levels on postnatal day 1 or 3 compared with spontaneous vaginal delivery, noting only a mild increase on day 2 (15). Abdelgader also reported no significant association between oxytocin infusion and serum bilirubin levels (16). Such inconsistencies may arise from several factors, such as study design and population differences (e.g., small-sample or cross-sectional studies with limited statistical power vs. our retrospective cohort with larger sample size and multivariable adjustment), variation in oxytocin administration protocols (total dose, duration, and infusion rate) across centers, differences in jaundice definitions and measurement time points (our study used AAP criteria, ≥12 mg/dL, focusing on the entire neonatal period), and inconsistent adjustment for maternal and perinatal confounders, including gestational age, delivery mode, and complications. These factors may either mask or exaggerate the true association between oxytocin and neonatal hyperbilirubinemia.

RCS-based dose–response analysis demonstrated that oxytocin doses below the median (∼14.8 IU) were not associated with significant changes in hyperbilirubinemia risk, whereas doses above this threshold showed a nonlinear increase. At 20 IU, the OR was 1.32, and at 25 IU, the OR rose to 1.56, indicating a significant risk increase with high-dose exposure. This finding provides a quantitative reference for safe clinical oxytocin use, suggesting that doses above the median should be closely monitored for neonatal serum bilirubin levels. Mechanistically, high-dose oxytocin enhances contraction intensity and frequency, potentially causing mild fetal hypoxia and reduced placental perfusion, impairing bilirubin clearance. It may also increase fetal red blood cell hemolysis or delay hepatic bilirubin metabolism, leading to elevated neonatal serum bilirubin. These observations are consistent with prior experimental and clinical studies on oxytocin-related placental hemodynamics and neonatal metabolic stress (6), reinforcing the plausibility of a causal relationship.

Interestingly, our analysis identified an inverse relationship between maternal BMI and neonatal hyperbilirubinemia. Although this finding may appear counter-intuitive, several physiological explanations are plausible. Higher maternal BMI is often associated with increased circulating lipid and glucose levels during pregnancy, which may enhance fetal hepatic maturation and bilirubin conjugation capacity through improved glycogen storage and glucuronosyltransferase activity. In contrast, lower maternal BMI may reflect reduced maternal energy reserves and micronutrient status, potentially impairing neonatal hepatic function and bilirubin clearance. Moreover, while women with higher BMI may experience longer labors, the total effective exposure to oxytocin in our cohort was not significantly correlated with BMI, suggesting that labor duration alone did not account for the observed association.

In addition, the absence of a significant association between fetal birth weight and bilirubin levels may be explained by the relatively narrow range of birth weights in our population (mean 3.4 ± 0.4 kg). This homogeneity limits the ability to detect subtle correlations, and future studies with larger, more diverse samples could further examine this aspect.

## 5 Clinical implications and limitations

This study establishes a clear association between oxytocin dose and neonatal hyperbilirubinemia risk, identifying a potential risk threshold through dose stratification and dose–response analysis, thereby providing a quantitative guide for safe clinical use. The findings suggest that obstetricians should consider both oxytocin dose and neonatal risk when administering the drug, avoiding prolonged or high-dose exposure, particularly above ∼15 IU, and monitoring neonatal serum bilirubin closely in such cases to reduce the incidence of hyperbilirubinemia and related complications. Additionally, maternal BMI was negatively associated with neonatal hyperbilirubinemia, suggesting that maternal nutritional status may modulate neonatal bilirubin metabolism, which could inform prenatal management strategies.

Several limitations should be noted. First, this was a single-center retrospective study, potentially introducing selection bias, and the clinical decision factors for oxytocin use could not be fully captured. Second, although major obstetric variables were included in the analysis, detailed information on the second stage of labor duration and uterine contraction frequency or number was not consistently available in the medical records, precluding a more refined analysis of intrapartum dynamics. Third, data on prostaglandin use for labor initiation were incomplete, and subgroup analyses by mode of delivery (vaginal vs. cesarean section) were limited by sample size and data structure. These unrecorded variables may have residual confounding effects that warrant further investigation. Finally, neonatal genetic and metabolic differences were not considered, which may affect hyperbilirubinemia risk assessment.

Future multicenter prospective studies with comprehensive intrapartum monitoring and standardized documentation of labor stages, uterine activity, and pharmacologic induction methods are warranted to further clarify the impact of oxytocin dose on neonatal bilirubin metabolism and the underlying mechanisms.

## 6 Conclusion

This study systematically delineates the association between oxytocin dose and neonatal hyperbilirubinemia risk, revealing a dose-dependent nonlinear risk pattern. These findings provide empirical evidence to support individualized oxytocin use in clinical practice and offer a scientific basis for neonatal risk management and monitoring strategies.

## Data Availability

The original contributions presented in the study are included in the article/Supplementary Material, further inquiries can be directed to the corresponding author.
